# *Haemoproteus syrnii* in *Strix aluco* from France: morphology, stages of sporogony in a hippoboscid fly, molecular characterization and discussion on the identification of *Haemoproteus* species

**DOI:** 10.1051/parasite/2013031

**Published:** 2013-09-13

**Authors:** Grégory Karadjian, Marie-Pierre Puech, Linda Duval, Jean-Marc Chavatte, Georges Snounou, Irène Landau

**Affiliations:** 1 UMR 7245 MCAM MNHN CNRS, Muséum National d’Histoire Naturelle 61 rue Buffon CP 52 75231 Paris Cedex 05 France; 2 Hôpital de la faune sauvage des Garrigues et Cévennes – Clinique vétérinaire 19 avenue du Vigan 34190 Ganges France; 3 Malaria Reference Centre – National Public Health Laboratory, Ministry of Health, 9 Hospital Drive, Block C, #04-01, Sing Health Research Facilities 169612 Singapore; 4 Inserm, UMR-S 945 91 Bd de l’Hôpital 75013 Paris France; 5 Université Pierre et Marie Curie-Paris 6, Faculté de Médecine Pitié-Salpêtrière, CHU Pitié-Salpêtrière 91 Bd de l’Hôpital 75013 Paris France

**Keywords:** *Haemoproteus syrnii*, *Strix aluco*, Volutin granules, Pupiparous fly, *Haemoproteus* subgenera, Molecular identification

## Abstract

In France, *Haemoproteus syrnii* is frequently found in the Tawny Owl, *Strix aluco*. Additional and complementary features of this species, and in particular the characteristics of volutin, are presented. The authors consider the volutin granules as constant in a given species, and discuss their taxonomic value. These cytoplasmic inclusions appear early during the first stages of development of the gametocytes as an initial granule which multiplies as the parasite develops. They were reported in some species of *Haemoproteus* but are seldom considered as a specific character and described with precision. Sporogony from ookinete to apparently mature sporozoites appears to take place in a pupiparous hippoboscid (*Ornithomyia* sp.). One specimen was crushed between two slides and stained with Giemsa. Gametocytes of *H. syrnii*, many ookinetes, an immature oocyst and mature sporozoites were observed spread all over the smear. This would allow classifying this species in the *Haemoproteus* subgenus. We provide associated molecular data derived from the cyt b and cox 1 gene from this parasite. We discuss the problems of multiple infections and the difficulties in identifying *Haemoproteus* species and in deriving conclusions from sequences deposited in databases.

## Abstract (fr)

En France, *Haemoproteus syrnii* est un parasite fréquent des chouettes hulottes, *Strix aluco*. Le parasite est redécrit et les auteurs insistent particulièrement sur les caractères de la volutine, qu’ils considèrent comme constants dans une espèce donnée et dont ils discutent la valeur taxonomique. Ces inclusions cytoplasmiques apparaissent dès les premiers stades du développement du gamétocyte sous forme d’un granule initial qui se multiplie au cours de l’évolution du parasite. Elles ont été signalées chez certaines espèces d’*Haemoproteus* mais sont rarement considérées comme un caractère spécifique et décrites avec précision. La sporogonie semble se développer du stade ookinète au stade sporozoïte apparemment mûr chez une Hippoboscidae pupipare (*Ornithomyia* sp.). Chez un spécimen écrasé entre deux lames et coloré au Giemsa ont été observés des gamétocytes d’*H. syrnii*, de nombreux ookinètes, un oocyste immature et des sporozoïtes matures dispersés sur la lame. Ceci permettrait de classer l’espèce dans le sous-genre *Haemoproteus.* Des données moléculaires concernant le parasite sont présentées (cyt b et cox 1). Le polyparasitisme et les difficultés à identifier les espèces d’*Haemoproteus* ainsi qu’à interpréter les séquences déposées dans les banques de données sont discutées.

## Introduction

*Haemoproteus syrnii* (Mayer, 1910) [22] was since its original description recorded in 30 species of Strigiformes [31]. Bishop and Bennett [2], in a review of parasites of Strigiformes, redescribed *H. syrnii* in *Strix varia* from Oklahoma (USA) and synonymized many other species based on published studies and from blood smears deposited at the International Reference Center for Avian Haematozoa (IRCAH). Martinsen *et al*. [18] using blood samples provided by Prof. I. Paperna who collected them from *Strix seloputo* and *Ninox scutulata* sampled in Singapore, in which *H. syrnii* was identified, provided gene sequences from these parasites. The corresponding material from Ilan Paperna’s collection was later deposited at the Muséum National d’Histoire Naturelle in Paris (MNHN), where after study we were able to redescribe the species and distinguish it from *H. syrnii* (Karadjian *et al*., unpublished).

The genus *Haemoproteus* has been subdivided by Bennett *et al*. (1965) in two subgenera [1]: *Haemoproteus* and *Parahaemoproteus*, mainly on the basis of different vectors: hippoboscid flies for the first one and *Culicoides* for the second, and on morphological differences of the sporogonic stages.

In his book on avian Haemosporidia, Valkiūnas [31] considered that species of the subgenus *Haemoproteus* must be restricted to parasites of Columbiformes. Later on, he realized, as did other authors [15], that the breadth of hosts of this subgenus could be much larger than previously thought. Levin *et al*. [15] redescribed *Haemoproteus iwa* Work & Rameyer (1996) [36] in *Fregata minor* from the Galapagos, using morphological and molecular tools (cyt b). They provided evidence that *H. iwa* is phylogenetically closely related to *Haemoproteus columbae* (Kruse 1890) [14], and was very probably transmitted by hippoboscid flies; consequently, this parasite should be classified in the *Haemoproteus* subgenus.

In this article we present morphological observations that complement the original description of *H. syrnii* in *Strix aluco* and propose to consider the volutin grains as important morphological and biological characters for *Haemoproteus* spp. gametocytes. We also provide associated sequence data of cytochrome b (cyt b) and cytochrome c oxidase I (cox 1) from this parasite and we discuss the difficulties in attempting to associate a particular sequence to a taxon, and to establish meaningful comparisons with data deposited in GenBank. Finally, we discuss the parasite’s taxonomic status and the host range of subgenera *Haemoproteus* and *Parahaemoproteus*.

## Materials and methods

### Materials

The biological material from *S. aluco*, included in this study, originated from Émile Brumpt’s collection and two location sites in France (see Table 1):

- Émile Brumpt’s collection: Seven blood smears collected in 1934 at Richelieu (Indre et Loire).
- Hôpital de la Faune Sauvage des Garrigues et Cévennes (Hérault): 39 blood smears from 7 adults and 32 juvenile *S. aluco*. For molecular characterization, two blood samples (one EDTA tube and one blood spot) were withdrawn from the brachial vein of birds (163BF and 154ZI) that harboured single infections with *H. syrnii*. Samples were collected from June 2011 to January 2013.
- Centre Régional de Soins pour la Faune Sauvage, Pas-de-Calais: 6 blood smears from 5 adults and 1 juvenile bird. Samples were collected from October to December 2011.

**Table 1.** Bird specimens (*Strix aluco*) studied, details about sampling and collection, and level of infection with *Haemoproteus syrnii* (−: uninfected; + to +++, level of infection).

Geographical origins	Dates of sampling	Age of birds	Infection	Sampling numbers	Collection numbers
Hérault (34)	3/6/2011	Juvenile	−		
	3/6/2011	Juvenile	−		
	3/6/2011	Juvenile	−		
	3/6/2011	Juvenile	−		
	3/6/2011	Juvenile	−		
	3/6/2011	Juvenile	−		
	3/6/2011	Juvenile	−		
	3/6/2011	Juvenile	−		
	3/6/2011	Juvenile	−		
	3/6/2011	Juvenile	−		
	3/6/2011	Juvenile	−		
	3/6/2011	Juvenile	−		
	3/6/2011	Juvenile	−		
	3/6/2011	Adult	++	163 BF	PXIV 14
	3/6/2011	Juvenile	−		
	25/6/2011	Adult	+++	167 BF	PXIV 15
	7/5/2012	Adult	+++	168 BF	PXIV 51–52
	7/5/2012	Juvenile	+++	169 BF	PXIV 53
	7/5/2012	Adult	+++	170 BF	PXIV 54
	7/5/2012	Adult	+	171 BF	PXIV 55
	7/5/2012	Adult	+++	172 BF	PXIV 56
	7/6/2012	Juvenile	−	562 VM	
	7/6/2012	Juvenile	−	563 VM	
	7/6/2012	Juvenile	−	564 VM	
	7/6/2012	Juvenile	−	565 VM	
	7/6/2012	Juvenile	−	566 VM	
	7/6/2012	Juvenile	−	567 VM	
	7/6/2012	Juvenile	−	568 VM	
	7/6/2012	Juvenile	−	569 VM	
	7/6/2012	Juvenile	−	570 VM	
	7/6/2012	Juvenile	−	571 VM	
	7/6/2012	Juvenile	−	572 VM	
	7/6/2012	Juvenile	−	573 VM	
	7/6/2012	Juvenile	−	574 VM	
	7/6/2012	Juvenile	−	575 VM	
	7/6/2012	Juvenile	−	576 VM	
	7/6/2012	Juvenile	−	577 VM	
	7/6/2012	Juvenile	−	578 VM	
	11/01/2013	Adult	+	154 ZI	PXIV 125
Pas-de-Calais (62)	5/10/2011	Juvenile	−		
	5/10/2011	Adult	−		
	5/10/2011	Adult	+++	840 JM	PXIV 16
	5/10/2011	Adult	−		
	5/10/2011	Adult	−		
	7/12/2011	Adult	−		
Indre-et-Loire (37)	18/8/1934	Unknown	+++	193 YY	PXIV 7
	19/8/1934	Unknown	+++	194 YY	PXIV 8−9
	21/8/1934	Unknown	+++	196 YY	PXIV 10−11
	20/8/1934	Unknown	+++	195 YY	PXIV 11
	22/8/1934	Unknown	+++	197 YY	PXIV 12
	4/8/1934	Unknown	+++	166 BF	PXIV 13
	7/8/1934	Unknown	+++	666 LV	PXIV 16

### Slides

All the blood smears on which the redescription was based were deposited in the collection of the Muséum National d’Histoire Naturelle, Paris, France. The record numbers and the dates at which the smears were made can be found in Table 1. All the smears were fixed by absolute methanol prior to Giemsa staining (10% in phosphate-buffered solution pH = 7.4) during one hour. They were then covered by a coverslip that was mounted with Eukitt^®^ resin before examination under oil immersion. Pigment granules were examined and photographed in unstained methanol fixed blood smears.

Slides from *Strix seloputo* included in Paperna’s collection are deposited in the MNHN under number 176BF, PIX58-60.

### Molecular methods

Blood samples were extracted using DNA Qiagen Micro Kit following the manufacturer’s instruction handbook for whole blood and blood spot extraction.

Partial mitochondrial gene cyt b (750 bp) and cox 1 (1293 bp) amplifications of the samples 163BF and 154 ZI were done using specific primers and protocols from Duval *et al*. (2007) [5]. The PCR products were sequenced using PLAS3 and PLAS4 primers by Cogenics. The cyt b partial gene sequences obtained included the cyt b gene region proposed as a standard for DNA bar-coding system for avian *Haemoproteus* species [9].

Avian Haemosporidia cyt b sequences were retrieved from GenBank (http://www.ncbi.nlm.nih.gov) for phylogenetic reconstruction. Molecular phylogeny was performed with 346 bp of mitochondrial cyt b gene by using Maximum-Likelihood methods with GTR model and nodal robustness evaluated by non-parametric bootstrapping (100 replicates) [8] (Figure 36). The phylogenetic tree was rooted using avian *Leucocytozoon* parasites.

Sequences of cyt b and cox 1 were deposited in GenBank as KF279523 and KF279522, respectively. The genetic distance between the Martinsen *et al.* (2006) [18] sequences and the newly generated sequences from *S. aluco* was estimated by *p*-distance method based on cyt b and cox 1.

## Results

In all the materials we have examined, we could only observe gametocytes identifiable as *H. syrnii*, though a *Plasmodium,* a *Trypanosoma* and a few gametocytes from a *Leucocytozoon*, were also observed in some specimens.

### Morphological redescription of *Haemoproteus syrnii* in *Strix aluco*

The rings and very young gametocytes lie in an apical or subapical position and are rarely in contact with the red blood cell (RBC) nucleus or its membrane.

**Figures 1–16.**
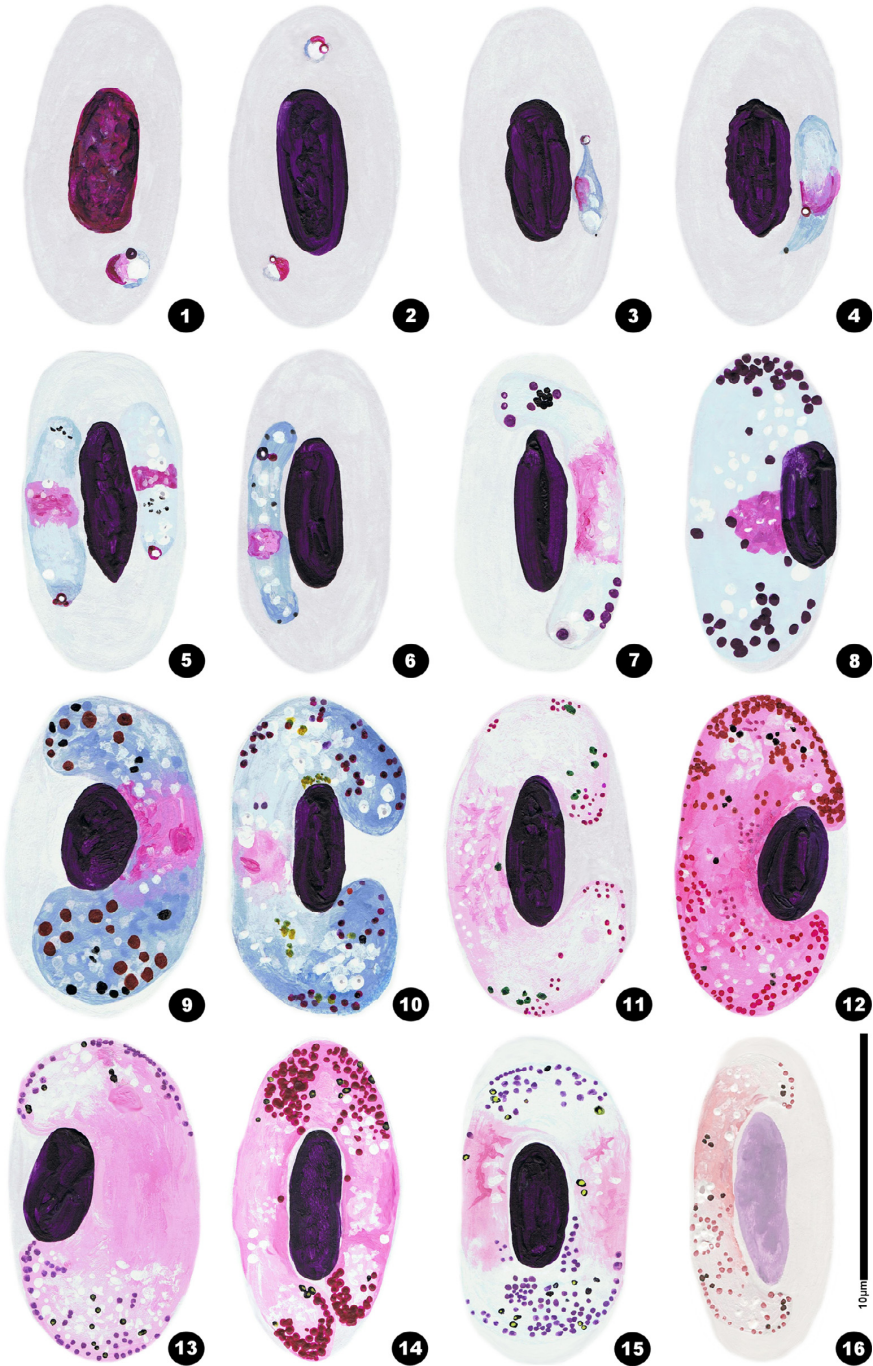
Drawings of erythrocytic stages of *Haemoproteus syrnii* in the blood of *Strix aluco*. 1–4: young trophozoites with an initial volutin granule; 5: two young gametocytes with 1 or 2 volutin granules attached to the initial granule; 6–7 young gametocytes; 8–10 mature macrogametocytes; 11, 13: mature microgametocytes; 12: old microgametocyte; 14: two microgametocytes within the same RBC; 15: a microgametocyte spread beneath the RBC nucleus; 16: microgametocyte with faded staining and where the volutin granules are still visible.

**Figures 17–31.**
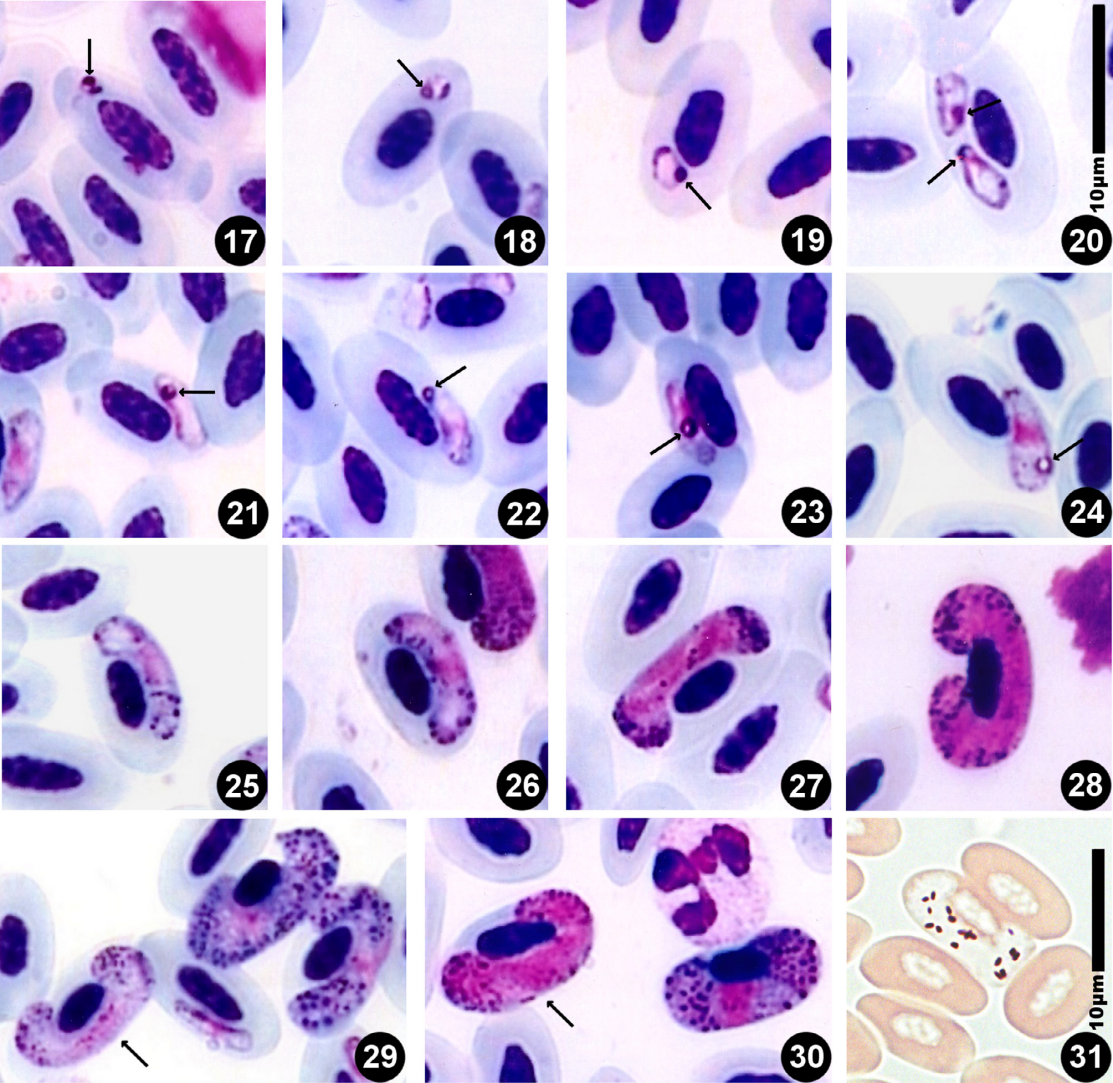
Microphotographs of *Haemoproteus syrnii* gametocytes in the blood of *Strix aluco*. 17–24: young gametocytes with the initial volutin granule (arrow); 25–26: immature gametocytes; 27–28: microgametocytes; 29: two macrogametocytes and a microgametocyte (arrow); 30: macrogametocyte and microgametocyte (arrow). Giemsa staining; 31: unstained smear: pigment of gametocyte.

The parasite is initially rounded or oval, and it comprises a cap-like nucleus, a large white vacuole and a first large granule of volutin deep garnet in colour with sometimes a less dark centre. Thereafter, the gametocyte elongates and becomes situated along the RBC nucleus. It has one end that is more peaked, even quite sharp, than the other. That first grain of volutin is found at this sharp end, or sometimes close to the nucleus. It is sometimes annular, a very characteristic feature (Figures 1–4, 17–24) that was previously observed by Mayer, in 1911, particularly in Figures 1 and 4 [21]. We have also observed cases where one or two granules appear to detach from the initial granule (Figure 5). At the next developmental stage, the gametocyte elongates further and the extremities become blunted (Figures 5–6, 22–25), the nucleus is median and the large vacuole from the younger stages has disappeared and small vacuoles are scattered in the cytoplasm (Figures 6, 23–25). Thereafter, it becomes loaded with volutin granules that progressively occupy the parasite’s ends (Figures 7, 26), and are particularly predominant in microgametocytes (Figures 11–14, 27, 28). In macrogametocytes, the nucleus is also median but the granules are distributed throughout the cytoplasm where many small vacuoles can be found (Figures 8–10, 29, 30). Finally, the parasite elongates yet further and its extremities curve around the RBC nucleus, though without encompassing it completely (Figures 9–13, 28–30). Generally one notes a thin space between the nucleus and the gametocyte. The opposing lateral border reaches the RBC membrane, but the curving ends leave a free space. Pigment granules are obscured by the volutin in stained slides and are described in unstained material (Figure 31): they are dispersed, black, elongated and of medium size (0.5–1.0 μm).

Volutin granules are distinctly finer in mature microgametocytes as compared to those in macrogametocytes. For both sexes the granules become smaller and more numerous as the gametocytes age. This ageing is accompanied by hyper-chromophilia; the chromatin is less granular and small very white vacuoles are seen throughout.

The RBC nucleus is central in young forms, whereas it appears more or less displaced in older or mature forms depending on the way the gametocyte is positioned on the slide at the time the blood smear is drawn; in these mature or aged gametocytes the nucleus is generally on a plane above that of the parasites and it might be rounded and condensed.

Notes: Mayer noted the presence of the first volutin granule in the very young stages [22] though he interpreted it as a second nucleus (kern). Our observations, and in particular the appearance of other attached granules, lead us to consider it as the initial volutin granule.

### Sporogony in a hippoboscid fly (*Ornithomyia* sp.)

**Figures 32–35.**
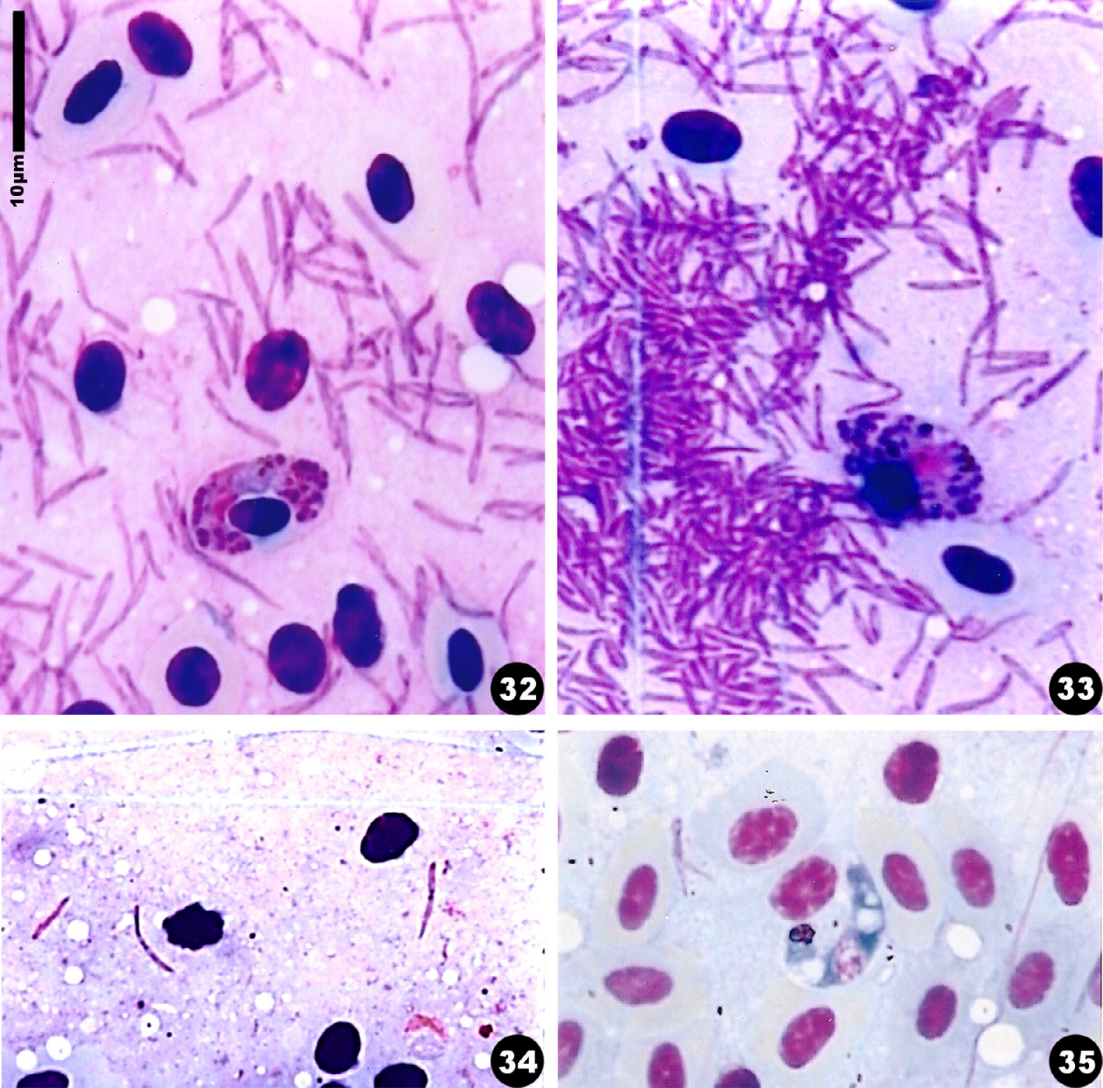
Microphotographs of developmental stages of *Haemoproteus syrnii* in the hippoboscid fly. 32: sporozoites issued from a burst oocyst and a gametocyte; 33: burst oocyst with sporozoites still attached to the cytomeres, and a gametocyte; 34: two mature sporozoites; 35: ookinetes. Giemsa staining.

In the smear obtained by squashing the hippoboscid fly we observed numerous elongated or rounded gametocytes (Figures 32, 33), and ookinetes (Figure 35). The shape and appearance of the gametocytes, including the presence of volutin, correspond to those of *H. syrnii*. Numerous sporozoites were dotted on the smear (Figures 32, 33). We have measured those that appeared to us to have reached maturity (Figure 34) (an average of 12 μm). They have a central nucleus formed by three grains, and a sharp extremity. At one place on the slide we noted a collection of smaller immature sporozoites radiating from centres (Figure 33), which probably represents a nearly mature oocyst that was burst by the squashing.

**Figure 36.**
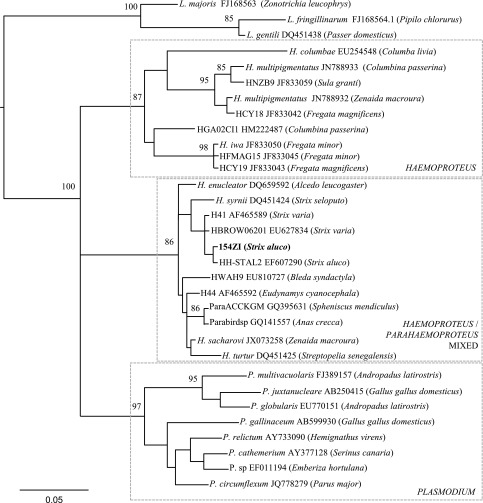
Maximum-likelihood phylogeny of mitochondrial cytochrome b lineages (346 bp) of avian *Plasmodium* spp. (8 sequences) and *Haemoproteus* spp. (21 sequences). Three sequences of *Leucocytozoon* spp. are used as outgroup. Bootstrap values > 70% are indicated near the nodes. GenBank accessions numbers and hosts (in parentheses) are indicated after the name of the parasite.

### Molecular characterizations and phylogenetic reconstruction

750 bp of cyt b and 1293 bp of cox 1 sequences obtained were associated with *H. syrnii* morphospecies identified and described in this paper. Alignment of these partial gene sequences with cyt b and cox 1 associated with *H. syrnii* published by Martinsen *et al.* [18] showed a molecular divergence of 3% for cyt b and 3.3% for cox 1.

Phylogenetically, *H. syrnii* is included in the group/clade that contains parasites from both the subgenera *Parahaemoproteus* and *Haemoproteus*.

## Discussion

### Prevalence

The great majority of the infected Tawny Owls were adults 6/10 (60%) while only 1/33 (3%) was a juvenile. However, most of the juveniles (31/33) had been examined in June. Although many had heavy infections with *Leucocytozoon* only one juvenile examined (07/05/2012) harboured *Haemoproteus* (Table 1).

### RBC nucleus

The morphology of the RBC and the position of the parasite with respect to the RBC nucleus are important characters for each species and they are generally noted in their descriptions. Many parasites surround the RBC nucleus completely or partially. In *H. syrnii*, the extremities of the parasite curve around the extremities of the RBC nucleus, though without encompassing it completely. In thin red blood films the parasite has a tendency to position itself on an inferior plane to that of the RBC nucleus, and as it reaches maturity to spread on the slide; by varying the focal point of the microscope one can perceive that the RBC nucleus is not on the same plane as the gametocyte. However, sometimes this appearance is misleading as it is interpreted in mature forms as that of a gametocyte that completely surrounds and engulfs the RBC nucleus (Figure 15).

### Volutin

Heavy production of volutin is observed in a number of *Haemoproteus* species that infect raptors, particularly species from two related families: Accipitridae and Strigidae. It was not noted in species infecting the Falconidae.

Bishop & Bennett (1989) [2] noted that the observation of volutin sometimes depends on the staining. Valkiūnas [31] considered that these cytoplasmic inclusion are of little interest to systematics: “The presence and amount of valutin in gametocytes of the majority of haemoproteid species are variable characters. Taken separately, as a rule, these characters cannot be used for the identification of *H. buteonis* and other species”. On the contrary, we consider it to be a constant characteristic, playing an important metabolic role, and to present distinct variations between the species. Émile Brumpt followed the infection in *S. aluco* over long periods (many weeks). We observed that volutin was always present in samples from his collection and that its characteristics remained invariant even when the smears were partially discoloured (Figure 16). Therefore, we consider these granules as important indicators of the parasite’s metabolism, and given the morphology and property that are characteristic for each species, as useful for parasite systematics.

The nature of the volutin grains observed in avian *Haemoproteus* has not been determined but it is probable that they represent acidocalcisomes (personal communication of Prof. Docampo). These are acidic cytoplasmic inclusions that are frequently observed in plants and in different parasites such as *Leishmania donovani* [28], *Toxoplasma gondii* [27], *Plasmodium berghei* [17], *Plasmodium falciparum* [16] and *Trypanosoma cruzi* [29, 30]. In the latter, the authors did detect a polyphosphate kinase and exopolyphosphatase activity in the acidocalcisome, and suggested that the organelle has a metabolic role that allows the parasite to adapt to environmental changes [29]. It would be interesting to investigate their role in some of the avian Haemoproteidae where they appear particularly early and with abundance, in contrast to numerous other species where they are not observed following Giemsa staining.

### Interpretation of the phylogenetic analysis

Phylogenetic analyses were based on partial sequences of the parasites’ gene coding for cytochrome b. Three well-supported groups emerge. The first group comprises *Plasmodium* species; the second group comprises *Haemoproteus* (*Haemoproteus*) species that include *H. columbae* and *H. multipigmentatus* Valkiūnas (2010) [35], both parasites of Columbiformes, and *H. iwa* Work & Rameyer (1996) [36], a parasite of Pelecaniformes, as well as other sequences that originate from Columbiformes and Pelecaniformes parasites that have not been identified at the species level; the third group contains *H. turtur* Covaleda (1950) [4], *sensu* Martinsen *et al*. (2006) [18], *H. sacharovi* (Novy & MacNeal 1904) [24] and *H. syrnii* (this study). The development of these last species in pupiparous dipterans justifies their classification in the *Haemoproteus* subgenus.

Another group, “*Strix* parasites” contains the *H. syrnii* (this study) and four other parasite sequences derived from other *Strix*. The *Haemoproteus* cyt b sequence identified by Martinsen *et al.* (2006) [18] diverges by 3% from the one we obtained. Two other *Haemoproteus* sequences found in parasites from *Strix varia* are identical to each other but differ from the *H. syrnii* sequence. Finally, a sequence from a parasite found in *S. aluco* in Germany [13] is identical to the one we obtained, though the author identified it as *H. noctuae* (Celli & San Felice 1891) [3], a species that, in contrast to *H. syrnii,* does not contain volutin. Thus, it is likely that the sequence attributed to *H. noctuae* is in fact from *H. syrnii,* a parasite that would have been missed during microscopic examination.

### Taxonomic status

The hippoboscid fly reached us squashed between two glass slides, which precluded the possibility to identify the species. The species most frequently observed on the birds of prey in Europe is *Ornithomyia avicularia* (Linnaeus, 1761). This pupipara is a ubiquitous ectoparasite recorded in a diversity of bird species [6, 11]. Indeed, this species retains well-developed wings that allow it to switch to another host with ease, when the one it is on is in distress or dies [20]. Through this behaviour, this insect could play an important role as a vector of haemoproteids within bird nests, colonies or dormitories. In our study, the hippoboscid fly was collected engorged with the blood of a *S. aluco* that was infected by *H. syrnii* and to which it was attached. Some observations show that *Haemoproteus* may develop until the oocyst stage in an abnormal host however they do not reach the sporozoite stage as seen in our infected Hippoboscid fly [34].

We consider it most likely that the complete development of the parasite that we observed in the insect, i.e. the mature sporozoite stage, belongs to *H. syrnii*. We thus consider, pending confirmation, that this suggests that this species should be classified within the subgenus *Haemoproteus*.

### Problems of species identification

Many natural infections are a mixture of different species. Ancient authors considered that each host harboured a single species and described morphological differences that were attributed to the variability of the same species. Molecular tools may sometimes detect the presence of more than one taxon. However, often only one of the species present in the blood is revealed and it is not always the predominant one. It could even be one that was overlooked when examining the slides with a microscope.

Valkiūnas *et al.* pointed out this bias [32], and also recommended great caution when comparing new sequences with those registered in GenBank which were not properly identified. They also considered that it is essential to associate the morphological description to the sequence data.

In several cases there are important discrepancies between the morphology in the original descriptions and the redescriptions by subsequent authors, and the sequences deposited in GenBank which are of uncertain identification.

An example of misidentifications relevant to our work is the following. In 2008, Paperna [25] succinctly described a “*Haemoproteus syrnii”* in *Strix seloputo* and *Ninox scutulata* from Singapore. A specimen of blood was sent to USA [19] for molecular analysis and the blood smears were deposited in the collection of the Muséum National d’Histoire Naturelle in Paris. It appeared that the parasite from Singapore [18, 19] and the parasite redescribed in this study are morphologically and molecularly two different species (Karadjian *et al*., unpublished data).

### The subgenera of *Haemoproteus*

Three species considered to belong to the subgenus *Haemoproteus* cluster with the *Parahaemoproteus* group in the various trees proposed [23, 33]: *H. syrnii*, *H. sacharovi* and *H. turtur*. *H. syrnii* and *H*. *saccharovi* seem to be single infections. We have examined more than 15 blood smears positive for *H. syrnii* without finding any evidence of a multiple species infection. Blood smears of *H. sacharovi* in *Zenaida macroura*, a parasite with a very characteristic morphology, were examined thoroughly by Valkiūnas *et al*. [33] and Krizanauskiene *et al*. [12] who concluded that the infection was single. In the case of *H. turtur* isolated by Prof Paperna from *S. senegalensis* and sequenced by Martinsen *et al.* [19] we cannot at present be sure that the infection was pure.

Krizanauskiene *et al*. [12] on the sole basis of the sequences obtained from the partial cyt b concluded that the *Haemoproteus* of birds should be classified in two monophyletic groups: subgenus *Haemoproteus* comprising *H. columbae* and all species clustering with it and subgenus *Parahaemoproteus* gathering all the other species [12]. They challenged the experimental conditions of transmission by pupiparans of *Haemoproteus saccharovi* by Huff [10] and of *H. turtur* by Rashdan [26], both species clustering with *Parahaemoproteus*.

We think that it is premature to establish a systematic classification of the haemoproteid species based only on the cyt b partial sequences and to draw conclusions from these data on the classification of the parasites which is based by definition on their the life cycle, i.e. vector, type of sporogony and type of tissue schizogony [1, 7].
